# Food and COVID-19: Preventive/Co-therapeutic Strategies Explored by Current Clinical Trials and in Silico Studies

**DOI:** 10.3390/foods9081036

**Published:** 2020-08-01

**Authors:** Giacomo Di Matteo, Mattia Spano, Michela Grosso, Andrea Salvo, Cinzia Ingallina, Mariateresa Russo, Alberto Ritieni, Luisa Mannina

**Affiliations:** 1 Laboratory of Food Chemistry, Department of Chemistry and Technologies of Drugs, Sapienza University of Rome, 00185 Rome, Italy; giacomo.dimatteo@uniroma1.it (G.D.M.); mattia.spano@uniroma1.it (M.S.); andrea.salvo@uniroma1.it (A.S.); cinzia.ingallina@uniroma1.it (C.I.); luisa.mannina@uniroma1.it (L.M.); 2 Department of Molecular Medicine and Medical Biotechnology, School of Medicine, University of Naples Federico II, CEINGE-Biotecnologie Avanzate, 80131 Naples, Italy; michela.grosso@unina.it; 3 Department of Agriculture, Food Chemistry Safety and Sensoromic Laboratory (FoCuSS Lab), University of Reggio Calabria, 89124 Reggio Calabria, Italy; mariateresa.russo@unirc.it; 4 Department of Pharmacy, School of Medicine, University of Naples Federico II, 80131 Naples, Italy; 5 UNESCO Chair of Health Education and Sustainable Development, University of Naples, 80131 Naples, Italy

**Keywords:** food, COVID-19, SARS-CoV-2, micronutrient Clinical trial, immune system, in silico study, protein interaction

## Abstract

Foods, food ingredients, and their balanced consumption are recognized to have an important role in achieving or maintaining a state of wellbeing by acting as carriers of functional components and bioactive molecules. However, the potential contribution of foods to consumers’ health has so far only been partially exploited. The rapidly evolving scenario of the coronavirus disease 2019 (COVID-19) pandemic is stimulating profound reflection on the relationships between food and the etiological agent, the severe acute respiratory syndrome coronavirus 2 (SARS-CoV-2). Here, the status of knowledge regarding food as a possible defense/co-therapeutic strategy against the SARS-CoV-2 coronavirus is considered through the discussion of two main current lines of research. One line of research relates to the role of micronutrients, food components, and diets in the strengthening of the immune system through clinical trials; formulations could be developed as immune system enhancers or as co-adjuvants in therapies. The other line of research relates to investigation of the chemical interactions that specific food compounds can have with host or virus targets so as to interfere with the viral infective cycle of SARS-CoV-2. This line requires, as a first step, an in silico evaluation to discover lead compounds, which may be further developed through drug-design studies, in vitro and in vivo tests, and, finally, clinical trials to obtain therapeutic molecules. All of these promising strategies promote the role of food in preventive/co-therapeutic strategies to tackle the COVID-19 pandemic.

## 1. Introduction

Foods are complex systems containing macro- and micronutrients, as well as plant secondary metabolites, that can take part in biochemical processes and help to achieve or maintain a state of wellbeing [1,2,3]. In the past few months, the potential contribution of foods to addressing the coronavirus disease 2019 (COVID-19) pandemic has become a highly debated topic, fueled by misleading fake news that is not supported by scientific results [4]. 

Coronaviruses (CoVs) are positive-sense single-stranded RNA viruses (Group IV of the Baltimore classification). CoVs are part of the Coronaviridae family and fall into the Orthocoronavirinae subfamily, which includes four genera: Alphacoronavirus, Betacoronavirus, Gammacoronavirus, and Deltacoronavirus [5,6]. Until 2019, six coronaviruses capable of infecting humans were known. Two of these, both from the Betacoronavirus group, caused major epidemics characterized by fatal respiratory disease: SARS-CoV, responsible for severe acute respiratory syndrome (SARS) in 2002 in the province of Guangdong in China; and MERS-CoV, responsible for Middle-East respiratory syndrome (MERS) in 2012 in the Kingdom of Saudi Arabia [7]. Recently, a new CoV, called severe acute respiratory syndrome coronavirus 2 (SARS-CoV-2) [8], was responsible for the outbreak of the COVID-19 pandemic on 31 December 2019 in China [9]. The previous SARS and MERS outbreaks involved 29 and 27 countries, respectively, and, given that over 170 countries have been affected by the COVID-19 pandemic after only 3 months, the severity of the current infection is evident [10,11,12]. The most common symptoms and signs of SARS-CoV-2 infection are respiratory symptoms, fever, dry cough, fatigue, and dyspnea [13,14], whereas the clinical manifestations of infection range from asymptomatic to severe pneumonia. It is noteworthy that pneumonia, a lower respiratory tract infection, has occupied the first position in the communicable disease rank and the fourth position in the top ten causes of death globally [15].

Although the design and production of a vaccine and its safe use represent the best solution to control the pandemic, to date, no pharmaceutical treatments or vaccines are available for COVID-19 [16]. However, unlike bacterial infections, viral diseases are often not treated with drugs because of latency, viral resistance, high mutation rates, and several frequent adverse side effects of treatment [17,18,19]. Therefore, eradicating viral diseases is rather challenging, and many viruses remain without preventive vaccines or effective antiviral treatments. Currently, only a few therapeutic protocols are available to reduce symptoms such as fever, pain, and impaired breath functionality, or to reduce diffuse microclots in the lungs [20].

Without a definitive effective treatment, a large number of research groups from all over the world are contributing their efforts to exploration of novel prevention or co-therapeutic approaches [21,22,23]. On 29 April 2020, 4 months after the outbreak started, 1580 articles in Scopus included the keyword "COVID-19", and by 29 May 2020 the number had reached 4091 articles.

**Figure 1:**
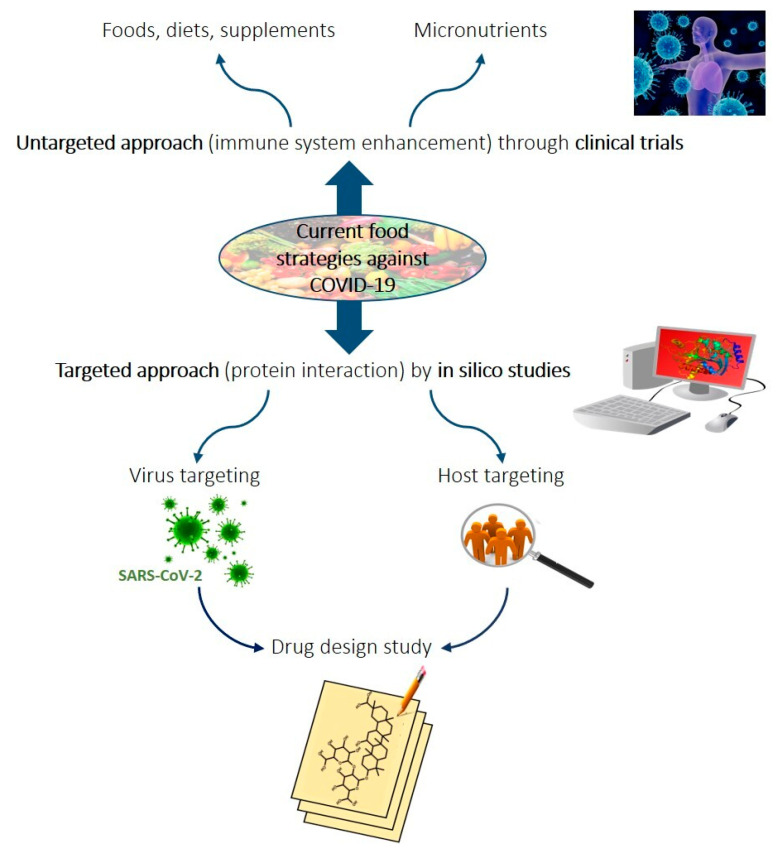
Scheme of current lines of research discussed in the present paper regarding food, food components, or diets used against coronavirus disease 2019 (COVID-19). The untargeted approach is based on immune system enhancement through food components and is being investigated in ongoing clinical trials, and the targeted approach is focused on the interaction between specific compounds and host or virus proteins and is being investigated by in silico studies.

In this review, the role of food and food nutrients in the prevention and intervention of pathology is thoroughly reviewed. Two ways to tackle COVID-19 with food are discussed, namely the untargeted approach through immune enhancement, which is currently being investigated in clinical trials, and the targeted approach through the interaction with host or virus proteins (Figure 1), which is being investigated by in silico studies. The strengthening of immune systems has been deeply investigated for previous viral infections; this approach is a part of several ongoing studies related to the current COVID-19 outbreak and is particularly interesting for elderly or immunodepressed populations [24,25]. The other line of research is focused on investigation of the structure [26], the mode of action [27], and the possible targets [28] of SARS-CoV-2. Food and natural products are in fact excellent sources for discovering novel antiviral tools, revealing new structure–activity relationships, and developing effective preventive/co-therapeutic strategies against viral infections [29,30,31].

## 2. Methodology

The clinical trials here reported were obtained using the Find Studies >New Search section in the U.S. National Library of Medicine Clinical Trials website [32]. The keywords “COVID-19” or “SARS-CoV-2” were searched in the “Condition or disease” field, with “All studies” selected in the “Status” field. Using these criteria with the two keywords, over 1800 studies were found on 24 May 2020. The twenty-three studies reported herein (see Table 1 and Supplementary Table S1) were selected by manually choosing only trials regarding food or nutrient topics and excluding all others in order to offer a comprehensive view of the current clinical trials on COVID-19 related to food as of 24 May 2020.

The in silico studies reported herein were obtained using the PubMed, Scopus, and Google Scholar databases. The keywords “in silico” or “docking” were paired with “SARS-CoV-2” or “COVID-19” to obtain articles published as of 4 July 2020. No language restriction was imposed. Some of the most relevant articles regarding food components were selected manually to give an overview of the current in silico studies on COVID-19 related to food. The selected articles are shown in Table 2.

## 3. Food as an Immune System Enhancer in Current Clinical Trials

The intake of some foods or food supplements can be extremely important for strengthening the immune system [33,34] and preventing the onset of pathologies such as respiratory infections. This aspect may represent a new frontier in the field of functional foods and an important resource for elderly people, as well as for immunosuppressed individuals or high-risk communities such as those in hospitals or retirement homes. An appropriate intake of micronutrients is necessary at every stage of growth, and particularly in some physiological conditions (older age, severe nutritional deficiencies, and stressful situations). Vitamins and minerals take part in many human biological and biochemical processes and are involved in immune system fortification (reinforcement of epithelial barriers, activation of immune cells, and enhancement of cytokines and antibody production) [35] and the wellness state. The role of some micronutrients as supporting agents in the prevention and treatment of respiratory tract viral infection has been largely demonstrated [36,37].

In the case of COVID-19, some clinical trials investigating the effect of food components on COVID-19 prevention/treatment strategies are currently in progress (see Table 1). Other information regarding these studies is reported in Supplementary Table S1 in Supplementary Materials.

It is noteworthy that all of the studies are still ongoing, and that each study presents its own eligibility criteria. In particular, two studies (No. 3 and No. 8) specifically involve elderly patients (over 60 and over 70 years of age, respectively), whereas in the others, participants of different age ranges have been engaged. Three trials are focused on prevention (No. 5, 13, and 23, COVID-19-negative participants), whereas all the others have a co-therapeutic aim. In particular, in trial No. 5, the effect of a prophylactic treatment based on hydroxychloroquine, vitamin C, vitamin D, and zinc supplementations for 600 health workers of both sexes (18 years and older) at high risk of being infected by SARS-CoV-2 is reported.

In trial No. 13, 1500 people of both sexes (from 18 to 75 years old) both positive and negative for COVID-19 were enrolled. In particular the selected positive patients presented symptoms of acute respiratory tract infection (e.g. fever, cough, dyspnea) accompanied by computed tomography (CT) scan of the chest compatible with COVID-19 or by a COVID-19 positive test (polymerase chain reaction, PCR). The aim of the trial is to study the prophylactic and co-therapeutic effects of orally administered calcifediol supplementation.

Sixteen clinical trials relate to the supplementation of a single micronutrient or a micronutrient combination. Seven trials involve the co-therapeutic use of vitamin C (No. 1, 2, 7, 9, 10, 11, and 15) for different purposes, including symptom reduction, mortality reduction, increase in ventilator-free days, and prevention of COVID-19 progression. In clinical trial No. 1, vitamin C and zinc gluconate are being administered alone or in combination in 520 patients of both sexes (18 years and older, treated with the standard therapy) in order to evaluate the reduction of COVID-19 symptoms. In all other clinical trials on vitamin C, it is being administered alone. In clinical trial No. 2, 500 hospitalized COVID-19 patients with pneumonia (ranging from children to elderly patients, treated with standard therapy) are being treated with vitamin C intravenously to observe a possible reduction of mortality and symptoms. In clinical trial No. 7, 200 COVID-19 patients of both sexes (18 years and older, treated with standard therapy) with acute lung injury and hypoxemia have been recruited and are being treated with early infusion of vitamin C to evaluate reduction of the lung injury caused by SARS-CoV-2. In clinical trial No. 9, 140 SARS-CoV-2-infected patients of both sexes with severe acute respiratory infection (18 years and older, treated with the standard therapy) have been recruited to evaluate a coadjutant protocol based on daily intravenous infusion, with the aim of improving the prognosis of patients. In trial No. 10, the effect of vitamin C infusion is being evaluated in 20 COVID-19 patients with both mild and severe deoxygenation problems (18 years to 99 years) to evaluate the effect of vitamin C in reducing respiratory failure. Another clinical trial (No. 11) aims to study the effect of vitamin C supplementation, orally or intravenously administered, in addition to a comparative treatment (two drugs and three supplements), on 200 COVID-19 patients (18 years and older, both sexes), with the aim of reducing disease progression. Finally, in trial No. 15, 66 hospitalized COVID-19 patients of both sexes (18 years and older, treated with standard therapy) are being treated with vitamin C infusions to observe a possible improvement of health status.

The widespread use of vitamin C in the clinical trials considered is due to the well-recognized role of this micronutrient in the prevention and treatment of respiratory illness [38,39]. For instance, in a previous clinical case, the treatment of a respiratory syndrome with intravenous injection of ascorbic acid together with respiratory assistance turned out to be effective for the improvement of patient healing after 10 days of therapy [40]. 

Five studies (No. 4, 8, 12, 13, 14) concern the administration of vitamin D. In clinical trial No. 4, 200 COVID-19 patients of both sexes with mild symptoms (from 40 years to 70 years, treated with standard therapy) are receiving vitamin D supplements in order to improve their immune system and slow down the symptoms progression. In clinical trial No. 8, the potential role of two doses of vitamin D_3_ (standard and high doses) as a supportive agent for COVID-19 treatment in 260 elderly patients (70 years and older, treated with standard therapy) of both sexes is being evaluated. In trial No. 12, 64 COVID-19 patients of both sexes (17 years and older) are being treated with low or high doses of vitamin D to study their eventual regression of symptoms. Finally, in trial No. 14, 1008 COVID-19 patients of both sexes (from 18 years to 90 years, treated with standard therapy) are receiving calcifediol with the aim of decelerate the syndrome progression. In two clinical trials (No. 3 and 16), vitamin D is being administered together with other supporting agents. In particular, in trial No. 3, vitamin D and zinc gluconate are being administered to 3140 COVID-19 elderly patients of both sexes (60 years and older, treated with standard therapy) in order to evaluate the reduction of inflammatory reactions. In trial No.16, vitamin D is being administered together with aspirin to 1080 COVID-19 patients of both sexes (18 years and older, treated with standard therapy) in order to mitigate the prothrombotic state and reduce hospitalization rates. In this case, the widespread use of vitamin D in clinical trials is also supported by literature data reporting the effectiveness of this micronutrient in the reduction of respiratory infection incidence. For instance, in a study by Urashima et al., vitamin D was administered to 167 schoolchildren via daily supplementation of 1200 IU (International Units), whereas another 167 schoolchildren were treated with a placebo for four months [41]. At the end of the trial, lower incidences of seasonal influenza A and asthma attack events were observed in the schoolchildren treated with vitamin D_3_ supplementation. 

It is noteworthy that in several of the clinical trials described above (No. 1, 3, 5 and 6), zinc is a component of the micronutrient mixture. The role of zinc in improving the immune system in several diseases, including respiratory infections, has been previously demonstrated [42]. For instance, a study carried out on two groups of children, treated with either placebo or zinc, showed a lower incidence (48%) of respiratory viral infections in the children treated with the zinc supplement [43].

In other clinical trials, the supplementation of other foods, food components, or diets has been proposed. In particular, trial No. 17 aims to evaluate the efficacy of natural honey in reducing the symptoms of 1000 COVID-19 patients of both sexes (from 5 years to 75 years, treated with standard therapy). Similarly, in trial No. 18, the efficacy of natural honey and black cumin seeds in reducing COVID-19 symptoms of 30 patients of both sexes (18 years and older, treated with standard care) is being investigated. 

The efficacy of a ketogenic diet in mitigating COVID-19 symptoms is also being tested (No. 19). In particular, in this clinical study, 15 COVID-19 patients of both sexes (18 years to 80 years) with respiratory failure requiring intubation have been recruited in order to study the efficacy of a ketogenic diet (high-fat, low-carbohydrate, adequate-protein diet) in the reduction of respiratory problems. This trial is supported by data from previous studies on the efficacy of ketone bodies in reducing artificial ventilator duration [44] and inflammation events [45]. In trial No. 20, a supplement enriched in eicosapentaenoic acid, linolenic acid, and antioxidants is being administered to 30 patients (18 years to 65 years older, treated with standard therapy) of both sexes to preserve their nutritional status and to evaluate the improvement of their health state. The role of polysaccharide intake is also being investigated: in trial No. 21, resistant potato starch is being administrated to 1500 nonhospitalized patients of both sexes (19 years and older, treated with standard therapy) in order to evaluate the reduction of disease progression, whereas in trial No. 22, gum arabic is being administrated to 110 patients of both sexes (5 years to 90 years, treated with standard therapy) as an immunomodulator and anti-inflammatory agent. 

Finally, in trial No. 23, 50 participants (from 18 years and older, treated with standard therapy) of both sexes have been enrolled in order to study quercetin’s efficacy both as prophylaxis in COVID-19-negative participants and as treatment in COVID-19-positive patients.

In addition to the micronutrients, food components, and diets being used in the reported clinical trials, other micronutrients with a demonstrated modulation action on the immune system could also be useful in other immune-enhancing formulations to reduce the risks of respiratory tract infections in the case of COVID-19. For instance, the roles of vitamins A, B, and E in the reinforcement of the immune system are well ascertained. Vitamin A supplementation has been shown to reduce respiratory disease incidence only in subjects with malnutrition or, conversely, an increase in disease risks [46] in the case of normal nutritional intake [47,48]. A correlation between vitamin B deficiency and several diseases, including respiratory viral infections, has been observed [49]. In a clinical observational study on 1176 children with acute lower respiratory tract infections, a relationship between an increase in disease incidence (44%) and low vitamin B_9_ concentrations in serum was observed [50]. The effective role of vitamin E supplementation for the prevention of respiratory diseases is still debated, since it has not yet been demonstrated. However, in a clinical study, daily vitamin E supplementation improved the immune system activity of 33 elderly women and men with immune deficiencies; their immune system activity became comparable to that of the control healthy adult group [51]. 

Regarding minerals, low serum levels of Se are associated with higher risks of immune weakness. A clinical study reported that 83 patients with respiratory diseases presented low serum selenium levels compared with a control group [52]. A correlation between iron supplements in children and lower incidence of upper respiratory tract infections was highlighted by De Silva et al. [53]. 

Notably, an important aspect to consider in trying to understand the activities and the efficacy of bioactive compounds in food and food nutrients is their bioavailability. Many factors may affect bioavailability, such as: bioaccessibility from natural sources; food matrix effects from synergistic, additive, or antagonist interactions among complex matrix components; molecular structures; physicochemical conditions; and the general health condition, genetic profile, or previous diseases of the consumers [54,55]. The bioactive compounds present in a food may have greater or lesser bioavailability compared with supplements. However, it is important to underline that supplements are often administered at high concentrations to compensate for their low bioavailability, which is due to the absence of passive or active carriers that co-introduce bioactive compounds when they are present as food ingredients. In contrast, the bioaccessibility of some food supplement ingredients is very high because of the absence of interactions with food matrices that may interfere with the digestion process [56].

Finally, micronutrients and food compounds with other additional biological activities could be of interest for addressing other concerns related to COVID-19. For instance, vitamin E, garlic, selenium, fish oil, ginkgo biloba, ginger, and ginseng, all of which have anticoagulant activity [57,58], could be proposed as adjuvants in the thrombotic events that have been recently clinically correlated with SARS-CoV-2 disease progression in a high percentage of patients [59,60].

## 4. Food Compounds in Preliminary in Silico Studies 

Although a vaccine is the most suitable solution to counteract the COVID-19 pandemic, its large-scale availability still requires the assessment of its toxicity, side effects, and efficiency in the population before allowing clinical trials [61]. Therefore, in this context, it may be of great relevance that some food components have adequate effects to be worth assessing with a targeted approach as potential candidates for treatment.

To better understand the potential roles of foods and their components in the fight against SARS-CoV-2, a brief description of the potential molecular targets already identified in the infection cycle [62] is needed. Considering the genomic similarities between SARS-CoV-2 and both MERS-CoV (50%) and SARS-CoV (79%) [63,64], some common features can be outlined in order to identify possible key factors for both drug discovery and food implications. It is well established that during the adsorption stage, the spike S-protein domain on the virus envelope is involved in binding with the human host angiotensin-converting enzyme (ACE2) to attack respiratory cells [65] in a very similar manner to that of the previous SARS-CoV. However, the much higher infectivity of this virus compared with that of the previous SARS-CoV and MERS-CoV infections could be explained by the recent finding of a region potentially involved in sialic-acid binding, which regulates host-cell infection [66]. Once SARS-CoV-2 is able to enter the cell and use the host translational machinery, the replication stage starts by expressing 16 nonstructural proteins (NSPs). Although not all of these proteins are well characterized yet, some of them may represent a potential target. In particular, viral papain-like protease (PL^pro^) and main protease (M^pro^ or 3CL^pro^) (the COVID-19 virus M^pro^ is in the Protein Data Bank (PDB) with accession number 6LU7) enzymes that are responsible for the cleavage of several critical viral proteins are considered the Achille’s heels of SARS-CoV-2, showing little structural variation compared with their SARS-CoV counterparts [67]. The NSPs assemble into the replicase–transcriptase complex (RTC) and create a suitable environment for viral RNA synthesis. This process follows the translation and assembly of viral replicase complexes. SARS-CoV-2 then releases RNA into the host cell. Genomic RNA is translated into viral replicase polyproteins pp1a and pp1ab, which are then cleaved into small products by viral proteinases. Using a discontinuous transcription process, the polymerase produces a series of subgenomic mRNAs that are finally translated into key viral proteins. Viral proteins and genomic RNA are subsequently assembled into virions in the endoplasmic reticulum and Golgi apparatus, and then transported via vesicles and released from the cell. 

Once molecular targets have been identified, studies to characterize potential drug candidates can be conducted in silico [28]. If the selected active compounds have good absorption, distribution, metabolism, excretion, and toxicity (ADMET) properties and respect “Lipinski’s Rule of 5” (a series of four rules that suggest if a molecule can be properly orally administrated, if more than one rule is not respected the molecule is not suitable for oral administration), then in vitro and in vivo analyses are required in order to define their efficacy, safety, and toxicological profile. Finally, clinical trials will be necessary to investigate the potential therapeutic efficacy and tolerability of the selected molecules [68]. An important advantage of these approaches is the rapid assessment of efficiency and toxicity in cell lines or animal studies in order to optimize the final choice. This advantage is essential, considering the rapid infection spread and the virus’ lethality. 

The main recent in silico studies regarding the affinity of naturally-occurring compounds for either viral targets or host targets to counteract SARS-CoV-2 are discussed below. The selection of molecules as potential COVID-19 antagonists for in silico studies was based on their in vitro activity on other coronaviruses, as previously described. The compound affinity depends on the type and amount of bonding that occurs with the active site of the protein [69].

Many studies have been focused on the activity of compounds isolated from food sources, such as glycyrrhizin, glabridin, glycyrrhetinic acid, and many polyphenols, such as caffeic acid, resveratrol, kaempferol, curcumin, demetoxicurcumin, quercetin, catechin, epicatechin gallate, hesperetin, hesperidin, δ-viniferin, and myricitrin. The results described hereinafter are also summarized in Table 2. In particular, M^pro^ has become the main virus target studied in in silico studies, followed by the human ACE2 receptor, SARS-CoV-2 spike S protein and RNA polymerase. However, the examined molecules should also be tested against other human and/or virus targets, since some molecules, such as curcumin, hesperidin, catechin, and garlic and cinnamon compounds, have been shown to be potentially active against other targets as well. Multitarget antiviral molecules can have greater efficacy against SARS-CoV-2 pathogenesis than molecules targeting a single protein [70]. Several food compounds have been shown to be active against other coronaviruses (mainly SARS-CoV), whereas other molecules, such as demethoxycurcumin and epicatechingallate, have not been investigated against coronaviruses but are being investigated because of their chemical similarities to well-known antiviral analogs. Finally, mixtures of compounds derived from food matrices, such as garlic and cinnamon, were separated and then individually analyzed in silico.

Hereinafter, the in silico studies related to isolated molecules from food sources are discussed.

Glycyrrhizin, a triterpene saponin, is the main compound of licorice root (*Glycyrrhiza glabra)*, a perennial herb used in traditional Chinese medicine [91]. The role of glycyrrhizin has long been studied in other viral infections, including HIV-1 and hepatitis C virus, in in vitro studies [92,93]. Moreover, the effect of glycyrrhizin was also studied against the SARS-CoV virus. It was found that glycyrrhizin inhibits viral replication and early steps of the replication cycle (adsorption and entry), with an unclear mode of action [72]. On the basis of these results, the effects against SARS-CoV-2 of glycyrrhizin was studied [71], although only in an in silico study, and a potential interaction of glycyrrhizin with a binding site near the hydrophobic site of the human ACE2 receptor was reported.

The importance of licorice as a source of active compounds was also confirmed by a study on glabridin, another triterpene from licorice root, which was tested against SARS-CoV-2. The compound was shown to have a high binding affinity with M^pro^ through one electrostatic and five hydrophobic interactions [73].

Glycyrrhetinic acid, another well-known triterpene in licorice root derived from the hydrolysis of saponin glycyrrhizic acid, showed the highest binding activity, among 2906 molecules tested, with a pocket of the SARS-CoV-2 spike S-protein that contributes to inhibiting the interaction with the ACE2 protein. This binding is based on hydrophobic and polar interactions with the steroidal scaffold of glycyrrhetinic acid and further reinforced by many ion and hydrogen interactions. The ability of the most active compounds to prevent virus entry in the case of low viral load was further confirmed in an in vitro study [94].

Polyphenols are a very large class of compounds known not only for their antioxidant properties but also for several other biological activities (antitumor, antibacterial, antiviral) [95]. Here, some previous studies about polyphenol activity against respiratory viruses and the current evidence related to SARS-CoV-2 from in silico studies are discussed.

Caffeic acid is a phenolic acid widely present in a variety of foods (such as fruits, vegetables, coffee, and propolis) [96]. A recent in vitro study showed that caffeic acid had powerful antagonist activity against human coronavirus (HCoV) NL63 [75], inhibiting the virus’ interaction with the ACE2 receptor. The potential antagonistic activity of caffeic acid toward SARS-CoV-2 was highlighted in a recent molecular-docking study in which the binding capacities of some propolis compounds against the virus M^pro^ were investigated [74]. This study showed that caffeic acid and its phenethyl ester have a good affinity for the active site of the enzyme, making these molecules potential virus antagonists. In the same study, two other propolis polyphenolic compounds, chrysin and galangin, were defined as potential anti-SARS-CoV-2 agents.

Resveratrol is a stilbenoid compound that exists in cis- and trans-isomeric forms, with the second being the predominant and biologically more active form [97], present in many nutritional foods such as grapes, peanut, blueberry, bilberry, cranberry, purple grapes, and grape juice [98]. Resveratrol has shown activity against many respiratory tract viruses [99] and also against SARS-CoV in an in vitro study [77]. This molecule also demonstrated a high binding affinity and the highest selectivity for the ACE2 complex, compared with the other stilbenoid compounds tested in a recent in silico study [76].

Additionally, δ-viniferin, a dehydrodimer of resveratrol produced along with other stilbenoids by stressed grapevine leaves, was also tested in in silico studies against SARS-CoV-2 to combat the cough symptoms of SARS-CoV-2 infection. δ-Viniferin, present in red wine [100] and used as an antitussive treatment in Indian medicine, has shown potent antiviral activity against a variety of viruses [101]. δ-Viniferin has been proven to be a multitargeted antiviral molecule against SARS-CoV-2, with a high binding affinity to M^pro^ through many interactions, such as polar, Pi-Pi, Pi-sulfur, and van der Waals interactions. In addition, this molecule also showed a high binding affinity with the RNA-dependent RNA polymerase (RdRp) target and with the ACE2 receptor [89].

The same study demonstrated the potential inhibitor activity of myricitrin, another antitussive molecule from Indian medicine present in *Myrica esculenta*, against SARS-CoV-2. This molecule presents a high binding affinity with M^pro^ through Pi–alkyl, Pi–sulfur, and van der Waals interactions and hydrogen bonds and, in addition, with the RdRp target and the ACE2 protein [89]. The interest in this molecule was also due to previous in vitro studies on SARS [78,90].

In a recent molecular-docking study [91], different polyphenols present in food matrices, namely kaempferol, curcumin, demetoxicurcumin, quercetin, catechin, and epicatechigallate, were investigated as potential COVID-19 inhibitors, having shown inhibitory activity against SARS in in vitro studies [79,81,82,83]. These molecules showed a high binding affinity to COVID-19 M^pro^, with low binding energies and inhibition constants. Kaempferol, a flavonol mainly present in tea and some vegetables (e.g., spinach, broccoli, cabbage) [102], showed the highest activity.

In another molecular-docking study, curcumin, present in turmeric, and catechin, present in tea, were found to be good potential antagonists against the human ACE2 receptor and the spike S protein of SARS-CoV-2 [80], as a result of their strong interactions with their binding sites.

Finally, hesperetin, a flavonoid present in citrus pericarp and albedo (*Citrus aurantium*, *Citrus reticulata*), showed dose-dependent inhibition of SARS M^pro^ in a recent in vitro study [98]. In a recent in silico study, it was shown that hesperetin has the potential to inhibit the ACE2 receptor, suggesting that this molecule might bind ACE2 and might interfere with SARS-CoV-2 infection [71]. Furthermore, hesperidin, a hesperitin glycoside, showed the potential to inhibit many proteins related to SARS-CoV-2 by interfering with their viral cycle [85]. These results underline the importance of further studies regarding this molecule and its industrial extraction process from citrus peel.

The second part of this section is focused on in silico studies on compound mixtures derived from food matrices to find the components with the highest binding affinity.

Garlic is a foodstuff known in many cultures for a variety of pharmaceutical properties [103]. Since ancient times, essential oils obtained from foods or natural material have played significant roles in pharmaceutics for the presence of potential therapeutic agents [104]. Because of the high chemical diversity of secondary metabolites, the use of natural essential oils in order to tackle the current SARS-CoV-2 pandemic has been extensively investigated. Many studies have demonstrated that garlic extracts possess in vitro biological activities, including antiviral activity against respiratory viruses [105]. Recently, Thuy et al. [86] characterized garlic essential oils via GC–MS (Gas Chromatography Mass Spectrometry) analysis and docking simulation, finding 17 organosulfur compounds (representing 99.4% of the garlic’s essential oil composition) capable of simultaneously inhibiting both the ACE2 host receptor and SARS-CoV-2 M^pro^ through noncovalent binding interactions. In particular, allyl disulfide, allyl trisulfide, diallyl tetrasulfide and trisulfide, and 2-propenyl propyl, which represent the major compounds of garlic essential oils, accounting for 59% of the entire composition, showed the highest affinity toward M^pro^. Surprisingly, the same four compounds showed the highest affinity values with the lowest docking score energies toward the ACE2 receptor. The multitargeted activity of these compounds is very promising, blocking the SARS-CoV-2 at two levels; at the entry stage by interacting with the ACE2 host receptor, and during the replication and transcription steps by interacting with M^pro^. 

Cinnamon, a compound obtained from different plant species from the genus *Cinnamomum*, is a source of many antiviral compounds in traditional Indian medicine [106]. Cinnamon extracts showed moderate inhibitory activity in wild-type severe acute respiratory syndrome coronavirus (SARS-CoV) and HIV/SARS-CoV S pseudovirus infections [88]. In a preliminary in silico study, 48 isolated compounds across all cinnamon species were subjected to docking analysis with the spike S protein and M^pro^ of SARS-CoV-2. Teinufolin showed the highest binding affinity with M^pro^ through six hydrogen interactions and hydrophobic interactions with two amino acids, and pavettanin C1 showed the highest binding affinity with the spike S protein through nine hydrogen and hydrophobic interactions with four amino acids [87].

## 5. Conclusions 

The potential contribution of food to consumers’ health and wellbeing has so far been only partially exploited by food science. We expect that the pandemic health emergency and the urgent need to strengthen prevention and control strategies will contribute to the mobilization of research efforts in innovative topics including nutraceutical activities, food/drug interactions, improved bioavailability, and novel formulations of natural food components. Food-based approaches generally offer the advantage of reduced adverse side effects with respect to conventional pharmacology approaches.

A promising approach that involves food chemistry could be the formulation of food-based immune enhancers for COVID-19 patients to allow the proper intake of macro- and micronutrients and, at the same time, to help reduce infection severity. 

Although micronutrients are safe compounds with important preventive and co-therapeutic activity for the treatment of many viruses, potentially including SARS-CoV-2, it should be considered that these molecules act on our biological system, and therefore, it is important to take food supplements in the correct doses, following expert advice, and only when it is necessary. It has been widely demonstrated that excessive doses of vitamins, mainly lipophilic ones, or minerals can provoke side effects that are harmful to human health. As confirmed by the Food and Agriculture Organization of the United Nations (FAO), the main preventive strategy against COVID-19 disease is the consumption of a healthy and balanced diet [107], whereas the use of supplements should be recommended only when really necessary. 

In this context, the ongoing clinical trials discussed herein will give useful indications of the efficacy of the proposed protocols (compounds, dose, administration, etc.) and direct research toward new strategies. 

The other strategic approach reported herein involves the discovery of lead compounds from food matrices through in silico studies; selected compounds can then be developed in drug-design studies, evaluated further in vitro and in vivo studies, and finally tested in clinical trials. Therefore, the food-science community could provide the most challenging and qualified contributions to investigation of the role of food both in the normal context and in the health emergency phase as it stands today.

In conclusion, many eating habits and beliefs about the role of food in human health have been shaken like an earthquake. At the end of the two-phase emergency, all of the main institutions, governments, and international agencies involving food and health consumers and the entire food chain are expected to kick-start and modify the global approach to food and its consumption, production, or transformation. In the remote past, humans used food as unique medicine; food-processing industrialization has reduced the natural value of their components, and the excessive use of ultraprocessed food has also favored morbidity conditions such as obesity, diabetes, and so on, that make individuals more susceptible to infective diseases. Therefore, it is arguable that the COVID-19 pandemic may be the first of many other global health crises that could be further exacerbated by progressively weakened immune systems.

## Figures and Tables

**Table 1:** List of the current clinical trials related to food (micronutrients, foods/diets, and other supplements) used for the prevention/treatment of COVID-19 as of 24 May 2020 [15]. A brief description reporting the trials’ aims, enrolment, and interventions is also reported.

№.	Treatment	Brief Trial Description ^ⱡ^^,$,^*	
**Micronutrients**			
1	Vitamin C Zinc gluconate	Aim	Reduce symptom duration
		Enrolment	18 years and older, COVID-19-positive, 520 participants
		Intervention	- IG 1: vitamin C (8000 mg/day) - IG 2: zinc gluconate (50 mg/day) - IG 3: vitamin C (8000 mg/day) and zinc gluconate (50 mg/day) - CG: standard care Duration: 10 days
2	Vitamin C	Aim	Reduce mortality and secondary symptoms
		Enrolment	All ages, COVID-19-positive, 500 participants
		Intervention	- IG: intravenous vitamin C (10 g/not specified) Duration: not specified
3	Zinc gluconate Vitamin D3	Aim	Reduce inflammatory reaction, which worsens acute respiratory distress syndrome
		Enrolment	60 years and older, COVID-19-positive, 3140 participants ^¥^
		Intervention	- IG: zinc gluconate (30 mg/day) and vitamin D3 (2000 IU (International Units)/day) - CG: standard care Duration: 2 months
4	Vitamin D	Aim	Improve hard endpoints related to COVID-19 deleterious consequences
		Enrolment	40 years to 70 years, COVID-19-positive, 200 participants
		Intervention	- IG: vitamin D (25,000 UI/day) - CG: standard care Duration: not specified
5	Hydroxychloroquine Vitamin C Vitamin D Zinc	Aim	Determine whether the combined therapy prevents COVID-19 symptoms ^ⴕ^
		Enrolment	18 years and older, FM, COVID-19-negative: high-risk individuals, 600 participants
		Intervention	- IG: hydroxychloroquine, vitamin C, vitamin D, zinc (not specified) Duration: not specified
6	Hydroxychloroquine Azithromycin Vitamin C Vitamin D Zinc	Aim	Determine whether the combined therapy can treat COVID-19 infection
		Enrolment	18 years and older, COVID-19-positive, 600 participants
		Intervention	- IG: hydroxychloroquine, azithromycin, vitamin C, vitamin D, zinc (not specified) Duration: 24 weeks
7	Vitamin C	Aim	Increase ventilator-free days, acute-inflammation-free days and organ-failure-free days
		Enrolment	18 years and older, FM, COVID-19-positive, 200 participants
		Intervention	- IG: intravenous vitamin C (100 mg/kg/8 hours) - CG: standard care Duration: 3 days
8	Vitamin D3	Aim	Improve the prognosis of older patients
		Enrolment	70 years and older, FM, COVID-19-positive, 260 participants ^¥^
		Intervention	- IG: vitamin D3 (400,000 IU/day) - CG: standard dose of vitamin D3 (50,000 IU/day) Duration: not specified
9	Vitamin C	Aim	Improve the prognosis of patients
		Enrolment	18 years and older, COVID-19-positive, 140 participants
		Intervention	- IG: intravenous vitamin C (24 g/day) - CG: standard care Duration: 7 days
10	Vitamin C	Aim	Reduce the risk of respiratory failure requiring mechanical ventilation
		Enrolment	18 years to 99 years, FM, COVID-19-positive, 20 participants
		Intervention	- IG 1: intravenous vitamin C (50 mg/kg/6 h) (mild deoxygenation) - IG 2: intravenous vitamin C (50 mg/kg/6 h) (severe deoxygenation) Duration: 4 days
11	Vitamin C Active comparator treatment (hydroxychloroquine, azithromycin, zinc citrate, vitamin D3 and vitamin B12)	Aim	Prevent COVID-19 progression
		Enrolment	18 years and older, COVID-19-positive, 200 participants
		Intervention	- IG: vitamin C (200 mg/kg/day on day 1, 400 mg/kg/day from day 2) and active comparator treatment (hydroxychloroquine, azithromycin, zinc citrate, vitamin D3 and vitamin B12) - CG: active comparator treatment Duration: 7 days
12	Vitamin D2 Vitamin D3	Aim	Determine the efficacy of vitamin D in patients
		Enrolment	17 years and older, FM, COVID-19-positive, 64 participants
		Intervention	- IG 1: vitamin D2 (4 doses of 50000 IU in 3 weeks) - IG 2: vitamin D3 (1000 IU/day) Duration: 3 weeks
13	Calcifediol	Aim	Study the preventive and therapeutic effects of oral calcifediol ^ⴕ^
		Enrolment	18 years to 75 years, COVID-19-negative and at high risk of acquiring COVID-19, or at risk for its morbidity and mortality, 1500 participants
		Intervention	- IG: oral calcifediol (25 µg/day) - CG: standard care Duration: 2 months
14	Calcifediol	Aim	Reduce the development of COVID-19 and the worsening of the various syndrome phases
		Enrolment	18 years to 90 years, COVID-19-positive, 1008 participants
		Intervention	- IG: oral calcifediol (266 µg/12 h on day 1, 266 µg/day on days 3, 7, 14, 21, 28) - CG: standard care Duration: 28 days
15	Vitamin C	Aim	Evaluate the safety and efficacy of ascorbic acid infusions in COVID-19 treatment
		Enrolment	18 years and older, COVID-19-positive, 66 participants
		Intervention	- IG: vitamin C infusion (0.3 g/kg on day 0, 0.6 g/kg on day 1, 0.9 g/kg on days 3–5) - CG: standard care Duration: 6 days
16	Aspirin Vitamin D	Aim	Test the hypothesis that treatment with aspirin and vitamin D in COVID-19 can mitigate the prothrombotic state and reduce hospitalization rates
		Enrolment	18 years and older, FM, COVID-19-positive, 1080 participants
		Intervention	- IG 1: aspirin (81 mg/day) - IG 2: aspirin (81 mg/day) and vitamin D (50000 IU/week) - CG: standard care Duration: 2 weeks
**Foods/diets**			
17	Natural honey	Aim	Study the efficacy of natural honey in patient treatment
		Enrolment	5 years to 75 years, COVID-19-positive, 1000 participants
		Intervention	- IG: natural honey (1 g/kg/day) - CG: standard care Duration: 14 days
18	Natural honey Black cumin	Aim	Reduce COVID-19 symptoms
		Enrolment	18 years and older, COVID-19-positive, 30 participants
		Intervention	- IG: natural honey and black cumin (1 g/kg/day) - CG: standard care Duration: 14 days
19	Ketogenic diet	Aim	Improve gas exchange, reduce inflammation and duration of mechanical ventilation
		Enrolment	18 years to 80 years, COVID-19-positive, 15 participants
		Intervention	- IG: ketogenic diet - CG: standard care Duration: 2 days
**Other supplements**			
20	Nutritional supplement enriched in eicosapentaenoic acid, gamma linolenic acid, and antioxidants	Aim	Reduce COVID-19 severity with more preservation of the nutritional status
		Enrolment	18 years to 65 years, COVID-19-positive, 30 participants
		Intervention	- IG: oral nutrition supplement (ONS) enriched in eicosapentaenoic acid, gamma linolenic acid, and antioxidants - CG: isocaloric/isonutrigenous ONS Duration: 14 days
21	Resistant potato starch Nonresistant corn starch	Aim	Determine the efficacy of resistant potato starch in reducing the need for hospitalization
		Enrolment	19 years and older, COVID-19-positive, 1300 participants
		Intervention	- IG: resistant potato starch (20 g/12 h) - CG: nonresistant corn starch (20 g/12 h) Duration: 14 days
22	Gum arabic Pectin	Aim	Study the efficacy of gum arabic as an immunomodulator and anti-inflammatory agent
		Enrolment	5 years to 90 years, COVID-19-positive, 110 participants
		Intervention	- IG: gum arabic (30 g/day) - CG: pectin (1 g/day) Duration: 4 weeks for IG and 12 weeks for CG
23	Quercetin	Aim	Evaluate the possible role of quercetin on prophylaxis and treatment of COVID-19 ^ⴕ^
		Enrolment	18 years and older, COVID-19-negative and -positive, 50 participants
		Intervention	- IG 1: quercetin (1000 mg/day in COVID-19 patients) - IG 2: quercetin (500 mg/day in NO COVID-19 patients) - CG: no intervention (in NO COVID-19 patients) Duration: not specified

^ⱡ^ IG = intervention group; CG = control group. ^¥^ The trial is being carried out on elderly participants. ^ⴕ^ Studies with prevention aim. ^$^: All studies are ongoing. * Both sexes were recruited.

**Table 2:** List of in silico studies related to food components. Details include the specific compound, chemical class, food source, anti-COVID-19 target, and previous studied activity on human coronaviruses (HCoVs). ACE2, angiotensin-converting enzyme; M^pro^, main protease; SARS-CoV, severe acute respiratory syndrome coronavirus.

Compound	Chemical Class	Food Source	In Silico Anti-COVID-19 Target	Previous in Vitro Activity on Other HCoVs
Glycyrrhizin	Triterpene Saponin	Glycyrrhiza glabra	ACE2 [71]	SARS-CoV [72]
Glabridin	Isoflavane	Glycyrrhiza glabra	Spike S protein [73]	
Caffeic acid	Phenolic acid	Fruits, vegetables, coffee, propolis	M^pro^ [74]	NL63 [75]
Caffeic acid phenylethyl ester	Phenolic ester	Honey, propolis	M^pro^ [74]	
Chrysin	Flavone	Honey, propolis	M^pro^ [74]	
Galangin	Flavonol	Honey, propolis	M^pro^ [74]	
Resveratrol	Flavonol	Grapes, peanut, blueberry, bilberry, Cranberry	ACE2 [76]	SARS-CoV [77]
Kaempferol	Stilbenoid	Tea, spinach, broccoli, Cabbage	M^pro^ [78]	SARS-CoV [79]
Curcumin	Diarylheptanoid	Turmeric	M^pro^ [78] ACE2 [80] Spike S protein [80]	SARS-CoV [81]
Demethoxycurcumin	Diarylheptanoid	Turmeric	M^pro^ [78]	
Quercetin	Flavonol	Fruits, vegetables	M^pro^ [78]	SARS-CoV [82]
Catechin	Flavanol	Tea	M^pro^ [78] ACE2 [80] Spike S protein [80]	SARS-CoV [83]
Epicatechigallate	Flavanol	Tea	M^pro^ [78]	
Hesperetin	Flavanone	Citrus peel and albedo	ACE2 [71]	SARS-CoV [84]
Hesperidin	Flavanone	Citrus peel and albedo	M^pro^ [85] ACE2 [85] Spike S protein [85]	SARS-CoV [85]
diallyl tetrasulfide, trisulfide 2-propenyl propyl	Organosulfur	Garlic	M^pro^ [86] ACE2 [86]	
Tenufolin	Triterpene saponin	Cinnamomun verum	M^pro^ [87]	SARS-CoV [88]
Pavettanin C1	Lignin	Cinnamomun verum	Spike S protein [87]	SARS-CoV [88]
δ-Viniferin	Flavonol	Red wine	M^pro^ [89] ACE2 [89] RNA-dependent RNA polymerase [89]	
Myricitrin	Flavone	Myrica esculenta	M^pro^ [89] ACE2 [89]	SARS-CoV [78,90]

## Supplementary Materials

The following are available online at https://www.mdpi.com/2304-8158/9/8/1036/s1, Table S1. List of the current clinical trials related to food (micronutrients, foods/diets, and other supplements) used for the prevention/treatment of COVID-19 with NTC identifier number, study name, treatment, aim, number of participants, study responsible, phase, and start and estimated completion dates.
