# Supplementary material for: Food and COVID-19: Preventive/Co-therapeutic Strategies Explored by Current Clinical Trials and in Silico Studies
Source: Foods. 2020 Aug 1;9(8):1036. doi: 10.3390/foods9081036 (PMC7466271; doi:10.3390/foods9081036)
Supplement: Supplementary file 1 [file foods-09-01036-s001.pdf]

Table 1. List of the current clinical trials related to food (micronutrients, foods/diets and other supplements) used for the prevention/treatment of COVID-19 with NTC identifier number, study name, treatment, aim, number of participants, study responsible, phase, and start and estimated completion dates [1].

№	ClinicalTrials.gov Identifier (NTC number)[$]	Study Name	Treatment (Dose)/Duration[ł]	Aim	№ Participants	Study Responsible	Phase *	Start Date ‡—Estimated Completion
**Micronutrients**								
1	NCT04342728	Coronavirus 2019 (COVID-19)-using ascorbic acid and Zinc supplementation (COVIDAtoZ)	- IG 1: vitamin C (8000 mg/day)<br>- IG 2: zinc gluconate (50 mg/day)<br>- IG 3: vitamin C (8000 mg/day) and zinc gluconate (50 mg/day)<br>- CG: standard care<br>• Duration: 10 days	Reduce the duration of symptoms	520	Cleveland Clinic, Weston, US	NA	April 2020–April 2021
2	NCT04323514	Use of ascorbic acid in patient with COVID-19	- IG: intravenous vitamin C (10 g/not specified)<br>• Duration: not specified	Reduce the mortality and secondary symptoms	500	A.R.N.A.S. Civico - Di Cristina - Benfratelli Hospital, Palermo, Italy	NA	March 2020–March 2021
3	NCT04351490	Impact of zinc and vitamin D3 supplementation on the survival of aged patient infected with COVID-19 (ZnD3-CoVici)	- IG: zinc gluconate (30 mg/day) and vitamin D3 (2000 IU/day)<br>- CG: standard care<br>• Duration: 2 months	Reduce the inflammatory reaction, which worsens acute respiratory distress syndrome (ARDS)	3140[¥]	University Hospital, Lille, France	NA	April 2020–July 2020
4	NCT04334005	Vitamin D on prevention and	- IG: vitamin D (25,000 UI/day)<br>- CG: standard care<br>• Duration: not specified	Improve hard endpoints related to COVID-19	200	Universidad de Granada,	NA	April 2020–June 2020

		treatment of COVID-19 (COVITD-19)		deleterious consequences		Granada, Spain		
5	NCT04335084	A study of hydroxychloroquine, vitamin C, vitamin D, and zinc for the prevention of COVID-19 infection (HELPCOVID-19)	- IG: hydroxychloroquine, vitamin C, vitamin D, zinc (not specified)<br>• Duration: not specified	Determine whether the combined therapy prevents COVID-19 symptoms[illegible]	600	ProgenaBiome, Ventura, US	2	May 2020–August 2021
6	NCT04334512	A study of quintuple therapy to treat COVID-19 infection (HZDpaC)	- IG: hydroxychloroquine, azithromycin, vitamin C, vitamin D, zinc (not specified)<br>• Duration: 24 weeks	Determine whether the combined therapy can treat COVID-19 infection	600	ProgenaBiome, Ventura, US	2	May 2020–August 2021
7	NCT04344184	Early infusion vitamin C for treatment of novel COVID-19 acute lung injury (EVICT-CORONA-ALI)	- IG: intravenous vitamin C (100 mg/kg/8 hour)<br>- CG: standard care<br>• Duration: 3 days	Increase ventilator-free days, acute-inflammation-free days, and organ-failure-free days	200	Hunter Holmes Mcguire Veteran Affairs Medical Center, Richmond, US	2	June 2020–May 2021
8	NCT04344041	COvid-19 and vitamin D supplementation: a multicentre randomized controlled trial of high dose versus standard dose vitamin D3 in high-risk COVID-19 patient (CoVitTrial)	- IG: vitamin D3 (400,000 IU/day)<br>- CG: standard dose of vitamin D3 (50,000 IU/day)<br>• Duration: not specified	Improve the prognosis of older patients	260[¥]	University Hospital, Angers, France	3	April 2020–July 2020

9	NCT04264533	Vitamin C infusion for the treatment of severe 2019-nCiV infected pneumonia	- IG: intravenous vitamin C (24 g/day)<br>- CG: standard care<br>• Duration: 7 days	Improve the prognosis of patients with severe acute respiratory infection (SARI)	140	Zhongnan Hospital of Wuhan University, Whuan, China	2	February 2020–September 2020
10	NCT04357782	Administration of intravenous vitamin C in novel coronavirus infection (COVID-19) and decreased oxygenation (AVoCaDO)	- IG 1: intravenous vitamin C (50 mg/kg/6 h) (mild deoxygenation)<br>- IG 2: intravenous vitamin C (50 mg/kg/6 h) (severe deoxygenation)<br>• Duration: 4 days	Reduce the risk of respiratory failure requiring mechanical ventilation	20	Hunter Holmes Mcguire Veteran Affairs Medical Center, Richmond, US	1, 2	April 2020–August 2020
11	NCT04395768	International ALLIANCE Study of Therapies to Prevent Progression of COVID-19	- IG: vitamin C (200 mg/kg/day on Day 1, 400 mg/kg/day from Day 2) and active comparator treatment (hydroxychloroquine, azithromycin, zinc citrate, vitamin D3, and vitamin B12)<br>-CG: active comparator treatment<br>• Duration: 7 days	Prevent the progression of COVID-19	200	National Institute of Integrative Medicine, Melbourne Australia	2	May 2020–December 2021
12	NCT04385940	Vitamin D and COVID-19 Management	- IG 1: vitamin D2 (4 doses of 50,000 IU in 3 weeks)<br>- IG 2: vitamin D3 (1000 IU/day)<br>• Duration: 3 weeks	Determine the efficacy of vitamin D (daily low dose versus weekly high dose) in COVID-19 patients	64	University of Alberta, Edmonton, Canada	3	May 2020–December 2020

13	NCT04386850	Oral 25-hydroxyvitamin D3 and COVID-19	- IG: oral calcifediol (25 µg/day)<br>- CG: standard care<br>• Duration: 2 months	Study the preventive and therapeutic effects of oral calcifediol on COVID-19	1500	Tehran University of Medical Sciences, Tehran, Iran	2, 3	April 2020–March 2021
14	NCT04366908	Prevention and Treatment With Calcifediol of COVID-19 Induced Acute Respiratory Syndrome (COVIDIOL)	- IG: oral calcifediol (266 µg/12h on day 1, 266 µg/day on days 3, 7, 14, 21, 28)<br>- CG: standard care<br>• Duration: 28 days	Reduce the development of COVID-19 and the worsening of the various phases of the syndrome	1008	Maimónides Biomedical Research Institute of Córdoba, Córdoba, Spain	2	April 2020–August 2020
15	NCT04363216	Pharmacologic Ascorbic Acid as an Activator of Lymphocyte Signaling for COVID-19 Treatment	- IG: vitamin C infusion (0.3 g/kg on day 0, 0.6 g/kg on day 1, 0.9 g/kg on days 3-5)<br>- CG: standard care<br>• Duration: 6 days	Evaluate the safety and efficacy of ascorbic acid infusions in COVID-19 treatment	66	Thomas Jefferson University, Philadelphia, US	2	May 2020–May 2021
16	NCT04363840	The LEAD COVID-19 Trial: Low-risk, Early Aspirin and Vitamin D to Reduce COVID-19 Hospitalizations (LEAD COVID-19)	- IG 1: aspirin (81 mg/day)<br>- IG 2: aspirin (81 mg/day) and vitamin D (50000 IU/week)<br>- CG: standard care<br>• Duration: 2 weeks	Test the hypothesis that treatment with aspirin and vitamin D in COVID-19 can mitigate the prothrombotic state and reduce hospitalization rates	1080	Louisiana State University Health Sciences Center in New Orleans, New Orleans, US	2	May 2020–December 2020

**Foods/diets**								
17	NCT04323345	Efficacy of natural honey treatment in patient with novel coronavirus	- IG: natural honey (1 g/kg/day)<br>- CG: standard care<br>• Duration: 14 days	Study the efficacy of natural honey in the treatment of patients	1000	Misr University for Science and Technology, Cairo, Egypt	3	April 2020–January 2021
18	NCT04347382	Honey & Nigella Sativa-COVID-Pakistan Trial Against COVID-19 (HNS-COVID-PK)	- IG: natural honey and black cumin (1 g/kg/day)<br>- CG: standard care<br>• Duration: 14 days	Reduce the COVID-19 symptoms	30	Mayo Hospital Kingedward Medical University, Lahore, Pakistan	3	May 2020–July 2020
19	NCT04358835	Keto-diet for intubated critical care COVID-19 (KICC-COVID19)	- IG: ketogenic diet<br>- CG: standard care<br>• Duration: 2 days	Improve gas exchange, reduce inflammation and duration of mechanical ventilation	15	Johns Hopkins University, Baltimora, US	NA	April 2020–August 2020
**Other supplements**								
20	NCT04323228	Anti-inflammatory/Antioxidant oral nutrition supplementation in COVID-19 (ONSCOVID19)	- IG: oral nutrition supplement (ONS) enriched in eicosapentaenoic acid, gamma linolenic acid, and antioxidants<br>- CG: isocaloric/isonutrigenous ONS<br>• Duration: 14 days	Reduce COVID-19 severity with more preservation of the nutritional status	30	King Saud University, Riad, Saudi Arabia	4	May 2020–October 2020

21	NCT04342689	The role of resistant potato starch in COVID-19 infection	-IG: resistant potato starch (20 g/12 h)<br>-CG: nonresistant corn starch (20 g/12 h)<br>• Duration: 14 days	Determine the efficacy of resistant potato starch in reducing the need for hospitalization	1300	Yale University, New Haven, US	NA	June 2020–June 2021
22	NCT04381871	Potential Role of Gum Arabic as Immunomodulatory Agent Among COVID 19 Patients (GA&COVID19)	-IG: gum arabic (30 g/day)<br>-CG: pectin (1 g/day)<br>• Duration: 4 weeks for IG and 12 weeks for CG	Study the efficacy of gum arabic as an immunomodulator and anti-inflammatory agent among COVID-19 patients	110	Omdurman Teaching Hospital Khartoum, Omdurman, Sudan	2, 3	June 2020–September 2020
23	NCT04377789	Effect of Quercetin on Prophylaxis and Treatment of COVID-19	- IG 1: quercetin (1000 mg/day in COVID-19 patients)<br>- IG 2: quercetin (500 mg/day in NO COVID-19 patients)<br>- CG: no intervention (in NO COVID-19 patients)<br>• Duration: not specified	Evaluate the possible role of quercetin in prophylaxis and treatment of COVID-19 □	50	Kanuni Sultan Suleyman Training and Research Hospital, Istanbul, Turkey	NA	March 2020–August 2020

* NA = not applicable. ¥ The trial is carried out on elderly participants. □ Studies with a prevention aim. ǀ IG = intervention group; CG = control group. ‡: Data on the basis of which the first participant was enrolled in the clinical study or the date that the researchers think will be the study start date. $ All studies are ongoing.

References

1. U.S. National Library of Medicine ClinicalTrials.gov Available online: https://clinicaltrials.gov/ct2/home (accessed on May 24 2020).
